# Intensity of resistance training via self-reported history is critical in properly characterizing musculoskeletal health

**DOI:** 10.1186/s12891-020-03753-w

**Published:** 2020-11-10

**Authors:** Todd C. Shoepe, Joseph W. LaBrie, Grant T. Mello, Allison G. Leggett, Hawley C. Almstedt

**Affiliations:** 1 Health and Human Performance Laboratory, Department of Health and Human Sciences, Loyola Marymount University, Life Sciences Building 181, 1 LMU Drive, MS 8888, Los Angeles, CA 90045 USA; 2 Department of Psychology, Loyola Marymount University, Los Angeles, CA USA

**Keywords:** Muscle quality, BMD, Strength, Peak bone mass, Sarcopenia, Osteoporosis

## Abstract

**Background:**

Intensity of resistance training history might be omitted or poorly ascertained in prescreening or data questionnaires involving musculoskeletal health. Failure to identify history of high-versus low-intensity training may overlook higher effect sizes with higher intensities and therefore diminish the precision of statistical analysis with resistance training as a covariate and bias the confirmation of baseline homogeneity for experimental group designation. The purpose was to determine the degree to which a single question assessing participant history of resistance training intensity predicted differences in musculoskeletal health.

**Methods:**

In the first research aim, participants were separated into groups with a history (RT) and no history (NRT) of resistance training. The second research aim evaluated the history of resistance training *intensity* on muscular strength, lean mass, and bone mineral density (BMD), RT participants were reassigned into a low- (LIRT) or high-intensity resistance training group (HIRT). 83 males and 87 females (19.3 ± 0.6 yrs., 171.1 ± 9.9 cm, 67.1 ± 10.5 kg, 22.9 ± 2.8 BMI, 26.2 ± 7.2% body fat) completed handgrip dynamometry (HG) and dual-energy x-ray absorptiometry scans (DXA) for BMD and bone mineral-free lean mass (BFLM).

**Results:**

A 3-group method (NRT, LIRT, HIRT) reduced type-I error compared with the 2-group method (NRT, RT) in characterizing the likely effects of one’s history of resistance training. For the second aim, HIRT had significantly (*p* < 0.05) greater HG strength (76.2 ± 2.2 kg) and arm BFLM (6.10 ± 0.16 kg) than NRT (67.5 ± 1.3 kg; 4.96 ± 0.09 kg) and LIRT (69.7 ± 2.0 kg; 5.42 ± 0.14 kg) while also showing significantly lower muscle quality (HG/BFLM) than NRT (13.9 ± 0.2 vs. 12.9 ± 0.3). HIRT had greater BMD at all sites compared to NRT (whole body = 1.068 ± 0.008 vs. 1.120 ± 0.014; AP spine = 1.013 ± 0.011 vs. 1.059 ± 0.019; lateral spine = 0.785 ± 0.009 vs. 0.846 ± 0.016; femoral neck = 0.915 ± 0.013 vs. 0.970 ± 0.022; total hip = 1.016 ± 0.012 vs. 1.068 ± 0.021 g/cm^2^) while LIRT revealed no significant skeletal differences to NRT.

**Conclusions:**

Retrospective identification of high-intensity history of resistance training appears critical in characterizing musculoskeletal health and can be ascertained easily in as little as a single, standalone question. Both retrospective-questionnaire style investigations and pre-screening for potential participation in prospective research studies should include participant history of resistance training *intensity*.

## Background

“Dysmobility” has been offered as a possible label [1] for the dual systemic, progressive chronic diseases characterized by decreasing quantity and quality of bone and muscle individually known as osteoporosis and sarcopenia respectively. Forecasts predict more than 300 million people worldwide at risk for osteoporotic fracture by 2040 [2] and more than 200 million people will be affected by sarcopenia in 2050 [3]. Both conditions contribute to declines in quality of life, physical disability, chronic disease comorbidity, increased risk of mortality, and burden the global healthcare system with tremendous financial expense including direct U.S. costs due to sarcopenia approximating $18.5 billion as of 2000 [4] and direct E.U. costs of osteoporosis approximating €38 billion in 2010 [5].

Prevention efforts for both disease conditions involve increasing peak bone mass (PBM) and muscle mass (PMM) prior to inevitable age-related declines [6–9]. Muscle mass is correlated with muscle strength [10–13], and age-related normative strength curves follow a similar trajectory to PBM and PMM, whereby peak muscle strength occurs near age 35 [14, 15]. However, muscle mass has only revealed weak-to-moderate relationships with strength which brings increased value to the concept of muscle quality (MQ), a variable defined as the ratio of strength per unit of muscle. Independently, MQ has value in predicting disability, disease, mortality, and some cancers [16–20]. Nonetheless, with evidence linking muscle mass muscle strength to positive bone outcomes [21], along with the prediction that increasing peak bone mass by 10% across a population might reduce the risk of later fracture by as much as 50% [22], the relationships of musculoskeletal mass, quality, and strength are of interest to athletes, patients, and practitioners alike.

Exercise, and in particular resistance training, is one of the most important strategies for improving musculoskeletal mass across the lifespan. With desired bone and muscle outcomes noted as a result of resistance training at different stages in life [23–29], lifelong resistance training programming is recommended for muscle strength, muscle mass, and bone health development [23, 30–32] in position stands by the National Strength and Conditioning Association (NSCA) and the American College of Sports Medicine (ACSM). Research consensus suggests that the higher forces imparted on the skeleton resulting from high-intensity resistance training promote more favorable outcomes in bone development, accrual, and retention than low-intensity resistance training [23, 32, 33]. Furthermore, low-intensity (< 50% of one-repetition maximum or 1RM) appears to provide adequate stimulus for strength development in novice lifters while high-intensity (> 80% of 1RM) appear more effective with experienced lifters [25, 30, 34]. Conversely, while hypertrophy can be seen across a spectrum of intensities in novice lifters, moderate to low intensities (70–85% of RM) are recommended for muscular hypertrophy among experienced lifters [30, 35]. The summary of these findings is that intensity of resistance training critically influences expected differential muscular and bone outcomes related to the prevention of osteoporosis and sarcopenia.

The use of questionnaires in behavioral and biological science is ubiquitous due to their inexpensive, simple nature but they come at the disadvantage of being more subjective and therefore more prone to inaccuracy [36]. In assessing exercise variables, low levels of agreement have been observed between perceived and actual duration/volume of exercise in retrospective surveys [37], and discrepancies between actual and recommended intensity have been documented with resistance training specifically [30]. It is therefore suggested that when possible, physiological measurements should be acquired in parallel with self-reported data as corroborative evidence to determinize relationships between activity and physiological outcome [38]. Therefore, with the known importance of intensity differentiation on musculoskeletal outcomes, and to properly confirm participant use of subjective terms describing intensity (e.g. light, medium, and heavy exercise), concurrently documented physiological measures that are objectively measured can aid in validating participant recall of exercise intensity [39].

Studies that neglect cross-sectional or baseline participant history of resistance training history and more specifically, the *intensity* of past participant training may be critically introducing systematic error into the expected outcomes of their investigation. Secondarily, it is critical to expound our understanding of the role that resistance training intensity plays in the development of peak musculoskeletal mass to improve our preventative recommendations of dysmobility. Therefore, the twofold aims of this investigation were to 1) describe the effect of two different classification methods of reported resistance training intensity history on variables of musculoskeletal health and 2) characterize potential differences in muscle mass, strength, MQ, and BMD in young adults due to their self-reported history of resistance training intensity. It was hypothesized that a) grouping all resistance training history into one group would be much less accurate in predicting musculoskeletal health than the separation of groups based on low- and high-intensity, and b) that a reported history of high-intensity resistance training would be associated with superior musculoskeletal health.

## Methods

### Experimental approach to the problem

This retrospective, cross-sectional investigation was performed as part of a federally-funded study investigating bone health and alcohol consumption in collegiate students partially described previously [40]. Participants were initially recruited through announcement via courses, on-campus student-life events, and postings to social media and website platforms. In a single laboratory visit, participants were given health and physical activity questionnaires, performed a muscular strength assessment, and completed a body composition assessment. All research protocols were approved by the Institutional Review Board for the Protection of Human Subjects and written informed consent was provided by each participant prior to any data collection procedures.

**Fig. 1:**
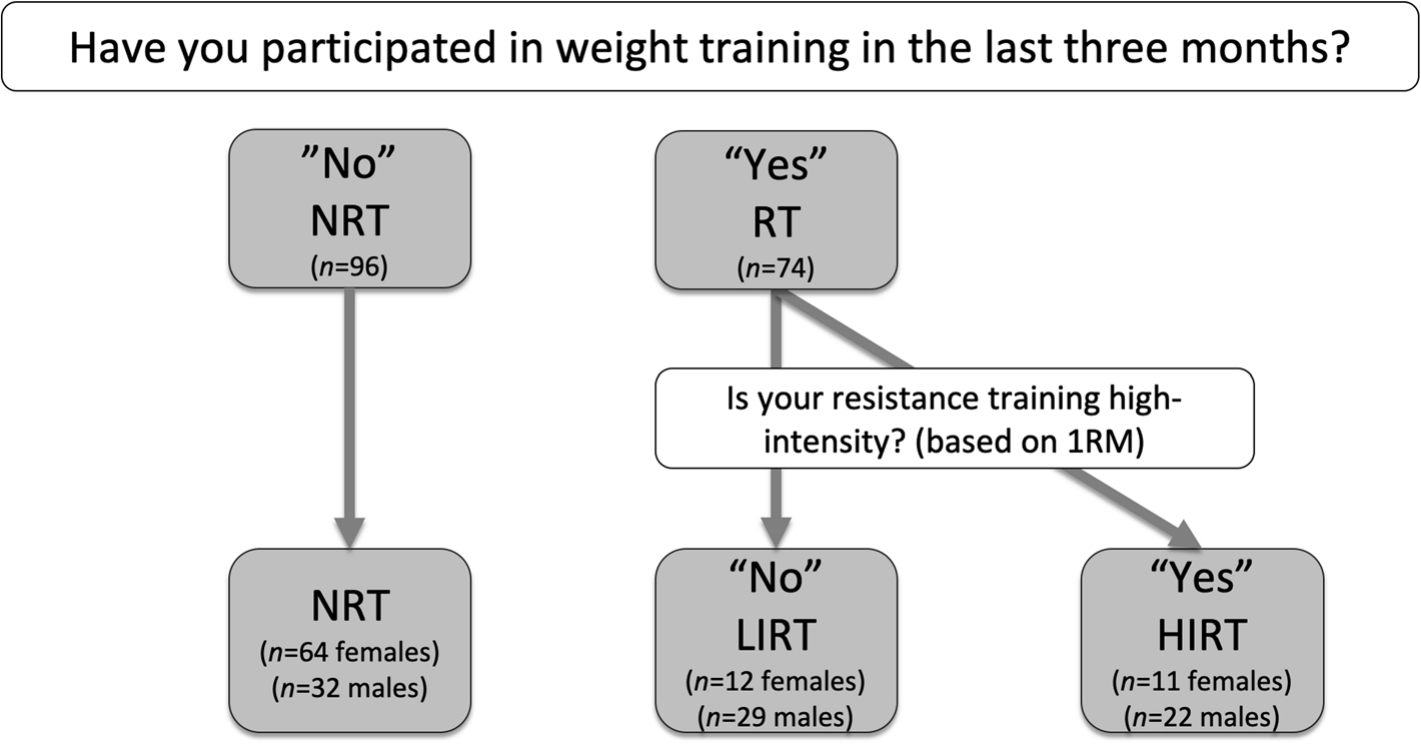
Group assignment flow chart

For this sub-investigation of the original study, two different research aims were investigated that were critically dependent upon assignment into either a two-group or three-group design (Fig. 1). The first research aim used self-reported recall to categorize participants into different groups based on resistance training history. In a comparison of two methods of categorization, a 2-group method separated participants into those having no history of resistance training (Group 1: NRT) or those with a history of resistance training (Group 2: RT). To determine the influence of intensity, a 3-group method categorized participants as having no history of resistance training (Group 1: NRT), a history of low-intensity resistance training (Group 2: LIRT), or a history of high-intensity resistance training (Group 3: HIRT).

### Participants

A total of 179 (89 = male, 90 = female) participants began the study although two individuals failed to complete all required testing protocols leaving 177 included in the preliminary analysis. Eligibility criteria for the study included status as a first- or second-year non-pregnant college student, and a self-reported BMI between 18.5–30 kg/m^2^. Descriptive anthropometric data are presented in Table 1.

**Table 1:** Participant descriptive values

2-group method	NRT	RT	NRT vs. RT ***p***			
*n*	96	74				
age (years)	19.2 ± 0.6	19.4 ± 0.6	**0.029**			
height (cm)	169.2 ± 8.5	173.6 ± 10.5	**0.003**			
body mass (kg)	64.9 ± 9.3	69.8 ± 11.4	**0.002**			
BMI (kg/m^2^)	22.7 ± 2.9	23.1 ± 2.8	0.332			
males/females	32/64	51/23				
**3-group method**	**NRT**	**LIRT**	**NRT vs. LIRT** ***p***	**HIRT**	**NRT vs. HIRT** ***p***	**LIRT vs. HIRT** ***p***
*n*	96	41		33		
age (years)	19.2 ± 0.6	19.5 ± 0.7	0.013*	19.3 ± 0.6	0.368	0.221
height (cm)	169.2 ± 8.5	173.8 ± 10.6	0.009*	173.2 ± 10.4	0.034*	0.804
body mass (kg)	64.9 ± 9.3	68.0 ± 11.0	0.103	72.1 ± 11.6	0.001*	0.092
BMI (kg/m^2^)	22.7 ± 2.9	22.5 ± 2.7	0.681	23.9 ± 2.5	0.032*	0.030*
males/females	32/64	29/12		22/11		

*p <0.05

All values presented as means ± SD

*NRT* non-resistance-trained, *RT* resistance-trained, *LIRT* low-intensity resistance-trained, *HIRT* high-intensity resistance-trained, *BMI* body mass index

### Procedures

Upon arriving at the Human Performance Laboratory, participants were given the Aerobic Center Longitudinal Study Physical Activity Questionnaire [41]. This was later used to calculate regular physical activity over the previous 3 months in metabolic equivalents (METs) with body mass in addition to stated duration of activities and intensity approximations according to published standards [41]. One item in this assessment requires respondents to identify whether they had participated in weight training at least once per week during the last 3 months. Participants who responded “no” to this question were assigned to the “no history” of resistance training group (NRT; *n* = 96; males = 32, females = 64). Original to this investigation included the follow-up prompt for those identifying a history of resistance training: “*Is your resistance training high-intensity? (i.e. based on 1RM*).” For the first research aim, all other participants entering “yes” were entered into the RT group (*n* = 74; males 51; females = 23). For the second research aim the RT group was divided into two separate groups where participants who responded “yes” to resistance training but did not use 1RM principles were assigned to a low-intensity (LIRT) group (*n* = 41; males = 29, females = 12) and finally, those identifying a training history based on 1RM principles were assigned to a high-intensity (HIRT) group (*n* = 33; males = 22, females = 11).

Daily energy, protein, vitamin D, and calcium intakes were assessed with the full-length 2014 Block Food Frequency Questionnaire (FFQ). This survey has been validated for retrospective dietary assessment over the previous year [42]. Following a brief portion size tutorial supported through the use of models and images, the survey required participants to approximate frequency of food consumption on a monthly, weekly, and daily basis from a list of foods developed from NHANES, based on the USDA Food and Nutrient Database. Calcium and vitamin D reported here were the result of summed dietary and supplemental values reported from the FFQ.

In minimal or athletic clothing sans shoes, height was assessed with a stadiometer (Seca Accu-Hite; Columbia, MD, USA) to the nearest 0.5 cm and an electronic scale was used to measure body mass to the nearest 0.1 kg (Tanita BWB-927A; Tokyo, Japan). Following positioning procedures that have been shown to produce scan-rescan coefficients of variation of less than 1% [43], whole body scans were performed for the assessment of body composition and segmental composition of the upper-extremities using dual-energy x-ray absorptiometry (DXA; Hologic Delphi A, Waltham, MA) and all scans were analyzed with Hologic Apex version 4.5 software. Whole body scans were also used for assessment of BMD (g/cm^2^) of the whole body (WB) while three additional regional scans allowed for densitometry assessment of the total hip (TH), femoral neck (FN), antero-posterior lumbar spine (AP), and lateral spine (LS). DXA has been identified as the gold standard in monitoring conditions of low BMD and as a pragmatic alternative to computed tomography (CT) and magnetic resonance imaging (MRI) in the assessment of muscle mass [44, 45].

Calibrations of the absorptiometer were performed prior to daily testing sessions during each of the testing periods. Scans were used to produce whole body and segmental extremity data for bone mineral-free lean mass (BFLM), fat mass, bone mineral content (BMC), and percent body fat. A prior check of internal consistency with this technician and system confirmed assessment reliability at greater than 99.0% with less than 1% coefficient of variation from repeated measurements of BMD measurements of the hip and spine from 20 volunteers of similar age to the study population whereby the participants were repositioned prior to the second scan.

As a proxy for total body strength [46], and following previously published testing recommendations [47], muscular strength was assessed with handgrip (HG) dynamometry whereby the same device was used for all assessments (Takei Physical Fitness Test Grip-D; Scientific Instruments Co. Ltd.; Niigata City, Japan). In order to normalize variations in grip score due to length-tension relationship of muscle [48], the handle of the dynamometer was adjusted prior to testing of each participant to about 90 degrees of flexion at the proximal interphalangeal joint resulting in the intermediate phalanx of the third digit lying parallel to the inner handle. Following a brief familiarization opportunity, participants were instructed to exert maximal effort for three trials lasting 3–5 s each alternating between the right and left hands. One minute of rest was provided between successive trials on each hand with the highest value for each hand used for data analysis. The variable of MQ was calculated as the sum of both maximal right- and left-hand grip scores divided by the sum of right and left arm bone-free lean mass (ABLFM) from the segmental DXA analysis. We chose ABFLM because deducting the non-contractile mineral mass from the segmental analysis provides a more sensitive measure of force production per muscle size. This was because hypothetically, two individuals with similar total arm mass, arm lean mass, and HG strength would demonstrate similar MQ. However, this finding would be subject to error in true muscle quality where in the case that these individuals had different bone mass values, the one with higher bone mass would have less contractile tissue and therefore a higher MQ.

### Statistical analysis

All statistical analyses were performed with the statistical package SPSS for MAC, version 24.0 (IBM Corp., Armonk, NY, USA) with a level of significance was set at *p* < 0.05. Descriptive values were assessed with multivariate analysis of variance (MANOVA). Mahalobnis difference, used to identify potential outliers in the primary outcome variables of HG, ABFLM, MQ, AP, LS, FN, TH, and WB revealed seven participants displaying outlier data to a probability of 0.0001 that were subsequently omitted. Normality was assessed with a Shapiro-Wilk’s test to a value of *p* < 0.05. Both HG (0.44, SE = 0.19) and ABFLM (0.49, SE = 0.19) were found to be positively skewed, while all other primary outcome variables were found to be normally distributed and analysis persisted based on minimum sample size recommendations to neglect violations of normalcy [49, 50]. All primary muscle and bone variables were confirmed to display homogeneity of variance with Levene’s test (*p* < 0.05). For the outcome analyses collapsed inclusion of males and females was justified through an examination of potential influence of sex on group differences examined through multivariate analysis of covariance (MANCOVA) with age, height, and calcium intake serving as covariates. Here, males and females exhibited either the same significant between-groups differences or trends for the same difference across training groups for primary dependent variables. Because of their well-established influence on BMD, this study covaried for the effects of participant height, age, and calcium intake [51]. Age was used as a covariate because the NRT group was significantly younger than others (See Table 1). Although the difference was less than 4 months in both cases, the participants were near the transition from adolescence to young adulthood when bone accrual is usually ongoing. Height was included due to its known scaling effects on muscle contractile function and BMD [52, 53] as well as to control for the disproportionate sex representation between groups.

#### Research aim #1

Subsequently, the primary outcome variables (*n* = 170) were assessed with one-way (with two groups) MANCOVA with height (*p* < 0.001), age (*p* = 0.41), and calcium intake (*p* = 0.23) as covariates with the following values for probabilities for observed power: HG (0.72), ABFLM (0.999), MQ (0.76), AP (0.17), LS (0.57), FN (0.64), TH (0.64), and WB (0.83). Observed effect sizes for HG, ABFLM, MQ, AP, LS FN, TH, and WB were determined for the 2-group method with Cohen’s *d* where the following categories were assigned based on previous recommendations [54] low effect = 0.2, moderate effect = 0.5, and large effect = 0.8.

#### Research aim #2

The primary outcome variables (*n* = 170) were assessed with one-way (with three groups) MANCOVA with height (*p* < 0.001), age (*p* = 0.41), and calcium intake (*p* = 0.23) as covariates with the following values for probabilities for observed power: HG (0.87), ABFLM (1.000), MQ (0.68), AP (0.51), LS (0.86), FN (0.56), TH (0.55), and WB (0.82). *Post-hoc* analysis was performed to examine between-group differences using Fisher’s Least Significant Difference (LSD). Observed effect sizes for HG, ABFLM, MQ, AP, LS FN, TH, and WB were determined for the 3-group method with Cohen’s *d* where the following categories were assigned based on previous recommendations [54] low effect = 0.2, moderate effect = 0.5, and large effect = 0.8. To test for the predictive influence of whole body composition, multiple stepwise linear regression analyses were performed separately for males and females with height, whole body mass, WFat, BFLM, and whole body fat percentage as potential predictors for each of AP, LS, FN, TH, and whole body BMD as dependent variables.

## Results

### Research aim #1

Participant anthropometric and descriptive values are provided in Table 1. Most importantly, the 2-group method where resistance training history was collapsed into a singular category, demonstrated differences between NRT and RT in all musculoskeletal variables except AP spine (Table 2). However, upon comparison to the 3-group model where RT was separated by intensity into two groups, there were no bone differences between low-intensity resistance training history whereas a history of high-intensity resistance training revealed significantly higher BMD for all bone variables (Table 3). Whole body composition, physical activity, and dietary intake comparisons are provided in Table 4.

**Table 2:** Musculoskeletal variables: 2-Groups

2-group method	NRT (95% CI)	RT (95% CI)	NRT vs. RT *p* (ES)
Combined right and left HG (kg)	67.5 ± 1.3 (64.9–70.0)	72.6 ± 1.5 (69.7–75.5)	0.011* (0.64)
Combined right and left arm BFLM (kg)	4.96 ± 0.09 (4.77–5.15)	5.73 ± 0.11 (5.51–5.94)	< 0.001* (0.89)
MQ	13.9 ± 0.2 (13.5–14.3)	13.1 ± 0.2 (12.6–13.5)	0.008* (0.60)
Anteroposterior spine BMD (g/cm^2^)	1.013 ± 0.011 (0.990–1.035)	1.031 ± 0.013 (1.005–1.057)	0.311 (0.24)
Lateral spine BMD (g/cm^2^)	0.785 ± 0.009 (0.767–0.803)	0.816 ± 0.011 (0.795–0.837)	0.033* (0.49)
Femoral neck BMD (g/cm^2^)	0.915 ± 0.013 (0.890–0.940)	0.961 ± 0.015 (0.932–0.990)	0.021* (0.50)
Total hip BMD (g/cm^2^)	1.016 ± 0.012 (0.992–1.041)	1.061 ± 0.014 (1.033–1.089)	0.021* (0.53)
Whole-body BMD (g/cm^2^)	1.068 ± 0.008 (1.052–1.085)	1.106 ± 0.010 (1.087–1.125)	0.013* (0.66)

*p <0.05

All values presented as estimated means ± SE from an ANCOVA adjusted for age, height, and total calcium intake

*95% CI* 95% confidence intervals, *NRT* non-resistance-trained, *RT* resistance-trained, *ES* observed effect size, *LIRT* low-intensity resistance-trained, *HIRT* high-intensity resistance-trained, *MQ* muscle quality, *BMD* bone mineral density, *HG* handgrip, *BFLM* bone-mineral free lean mass

**Table 3:** Musculoskeletal variables: 3-Groups

3-group method	NRT	LIRT	NRT vs. LIRT	HIRT	NRT vs. HIRT	LIRT vs. HIRT
	(95% CI)	(95% CI)	*p* (ES)	(95% CI)	*p* (ES)	*p* (ES)
Combined right and left HG (kg)	67.5 ± 1.3 (61.8–68.4)	69.7 ± 2.0 (68.0–78.1)	0.035* (0.48)	76.2 ± 2.2 (73.3–84.5)	0.001* (0.84)	0.028* (0.34)
Combined right and left arm BFLM (kg)	4.96 ± 0.09 (4.40–5.00)	5.42 ± 0.14 (5.32–6.23)	0.009* (0.73)	6.10 ± 0.16 (5.89–6.90)	< 0.001* (1.09)	0.002* (0.37)
MQ	13.9 ± 0.2 (13.6–14.5)	13.2 ± 0.3 (12.4–13.6)	0.066 (0.54)	12.9 ± 0.3 (12.0–13.4)	0.014* (0.67)	0.515 (0.13)
Anteroposterior spine BMD (g/cm^2^)	1.013 ± 0.011 (0.987–1.031)	1.007 ± 0.017 (0.978–1.045)	0.792 (0.3)	1.059 ± 0.019 (1.029–1.104)	0.041* (0.52)	0.046* (0.48)
Lateral spine BMD (g/cm^2^)	0.785 ± 0.009 (0.760–0.797)	0.791 ± 0.014 (0.770–0.827)	0.706 (0.02)	0.846 ± 0.016 (0.825–0.889)	0.001* (0.86)	0.010* (0.61)
Femoral neck BMD (g/cm^2^)	0.915 ± 0.013 (0.880–0.933)	0.954 ± 0.020 (0.922–1.002)	0.108 (0.41)	0.970 ± 0.022 (0.940–1.029)	0.032* (0.57)	0.566 (0.16)
Total hip BMD (g/cm^2^)	1.016 ± 0.012 (0.981–1.032)	1.055 ± 0.019 (1.028–1.106)	0.098 (0.45)	1.068 ± 0.021 (1.039–1.126)	0.036* (0.64)	0.625 (0.11)
Whole-body BMD (g/cm^2^)	1.068 ± 0.008 (1.041–1.077)	1.095 ± 0.013 (1.079–1.134)	0.087 (0.52)	1.120 ± 0.014 (1.103–1.165)	0.002* (0.85)	0.198 (0.28)

*p <0.05

All values presented as estimated means ± SE from an ANCOVA adjusted for age, height, and total calcium intake

*95% CI* 95% confidence intervals, *NRT* non-resistance-trained, *RT* resistance-trained, *ES* observed effect size, *LIRT* low-intensity resistance-trained, *HIRT* high-intensity resistance-trained, *MQ* muscle quality, *BMD* bone mineral density, *HG* handgrip, *BFLM* bone-mineral free lean mass

**Table 4:** Muscle, bone, physical activity, and nutrition

	NRT (95% CI)	LIRT (95% CI)	NRT vs. LIRT	HIRT (95% CI)	NRT vs. HIRT	LIRT vs. HIRT
			p		p	p
Whole body BMC (kg)	2.203 ± 0.329 (2.126–2.280)	2.424 ± 0.464 (2.306–2.542)	0.002*	2.565 ± 0.409 (2.434–2.696)	< 0.001*	0.116
Whole body fat mass (kg)	18.63 ± 5.53 (17.46–19.79)	15.93 ± 5.99 (14.15–17.70)	0.013*	16.98 ± 6.13 (15.00–18.96)	0.158	0.437
Whole body BFLM (kg)	44.70 ± 7.72 (42.97–46.42)	50.38 ± 9.66 (47.74–53.02)	< 0.001*	52.96 ± 9.45 (50.00–55.90)	< 0.001*	0.199
Whole body percentage body fat (%)	28.4 ± 6.6 (27.0–29.7)	23.2 ± 7.2 (21.1–25.3)	< 0.001*	23.3 ± 7.2 (21.0–25.7)	< 0.001*	0.934
Physical activity (MET•hrs•wk.^− 1^)	41.9 ± 54.7 (30.5–53.3)	55.6 ± 47.9 (38.2–73.1)	0.194	71.0 ± 70.3 (51.6–90.5)	0.012*	0.247
Caloric intake (kcals•d^− 1^)	1833.4 ± 839.7 (1645.7–2021.1)	2090.4 ± 1113.8 (1803.2–2377.6)	0.141	2363.7 ± 949.3 (2043.5–2683.8)	0.005*	0.212
Total calcium intake (mg•d^−1^)	988.9 ± 478.6 (891.9–1085.9)	1044.8 ± 477.9 (896.4–1193.2)	0.535	1236.8 ± 493.5 (1071.3–1402.2)	0.012*	0.090
Total vitamin D intake (μg•d^− 1^)	9.61 ± 18.87 (6.63–12.50)	8.24 ± 5.79 (3.68–12.80)	0.621	8.44 ± 6.56 (3.53–13.52)	0.695	0.955
Protein intake (g•d^− 1^)	69.5 ± 35.7 (61.5–77.5)	87.2 ± 48.1 (74.9–99.4)	0.018*	89.7 ± 39.7 (76.0–103.3)	0.013*	0.786
Relative protein intake (g•kg^− 1^•d^− 1^)	1.08 ± 0.56 (0.97–1.2)	1.25 ± 0.60 (1.08–1.44)	0.112	1.28 ± 0.62 (1.08–1.48)	0.099	0.878

*p <0.05

All values presented as estimated means ± SD

*95% CI* 95% confidence interval, *NRT* non-resistance-trained, *LIRT* low-intensity resistance-trained, *HIRT* high-intensity resistance-trained, *BMC* bone mineral content, *BFLM* bone-mineral free lean mass, *MET* metabolic equivalents

### Research aim #2

**Fig. 2:**
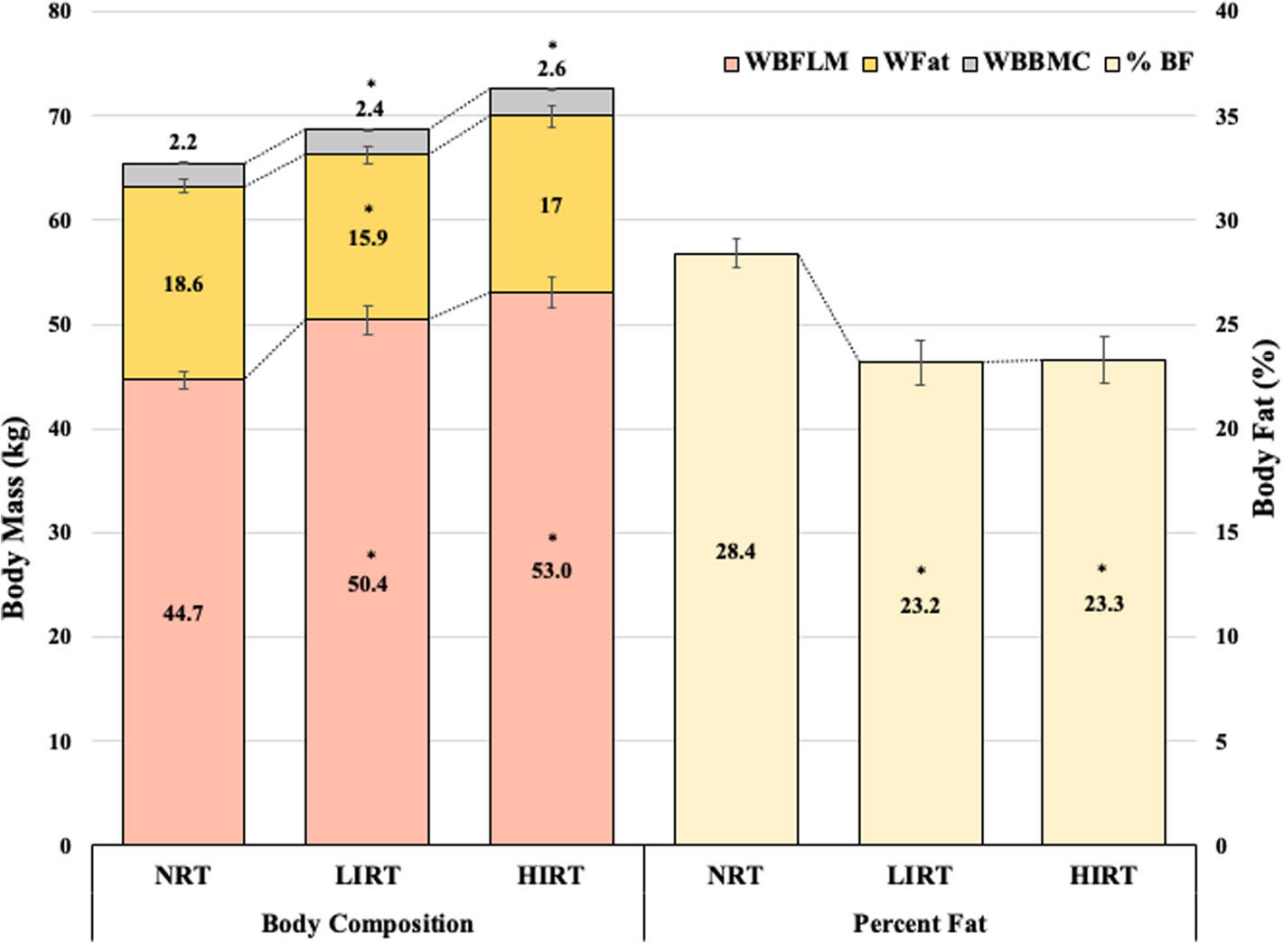
Whole body composition for research aim 2. All values presented as means. * = different than NRT (*p* < 0.05); NRT = non-resistance-trained; LIRT = low-intensity resistance-trained; HIRT = high-intensity resistance-trained; WBFLM = whole body bone-mineral-free lean mass; WFat = whole body fat mass; WBBMC = whole body bone mineral content

**Fig. 3:**
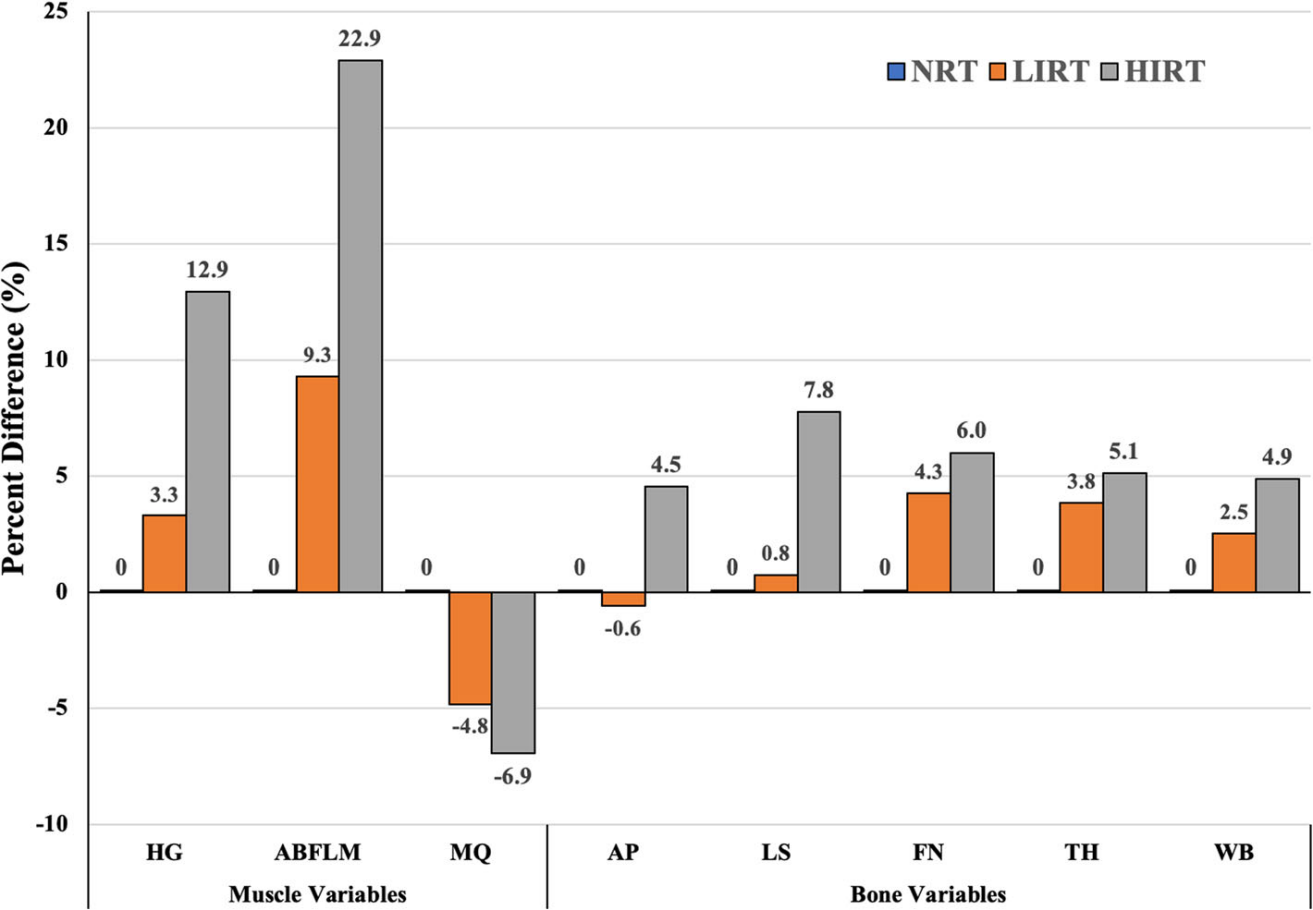
Between-group differences in musculoskeletal health. All values are presented as group mean percent differences from the non-resistance-trained group normalized to zero as a comparison. NRT = non-resistance-trained; LIRT = low-intensity resistance-trained; HIRT = high-intensity resistance-trained; HG = combined left and right maximum handgrip strength; ABFLM = combined right and left arm bone-mineral-free lean mass; MQ = muscle quality (HG/ABFLM); AP = anteroposterior bone mineral density; LS = lateral spine bone mineral density; FN = femoral neck bone mineral density; TH = total hip bone mineral density; WB = whole body bone mineral density

Both intensity groups demonstrated differences for all whole body composition values compared to NRT (with the exception of total body fat mass of HIRT) (Fig. 2). However, while there were no dietary or activity differences found between the LIRT and NRT groups, the HIRT group were more active, consumed more calories, had higher calcium intakes, and consumed higher daily absolute protein. Regression results showed WBFLM to be the only included variable or the highest variable for each bone site (with *r*^*2*^ ranging from 11 to 29%) in both males and females except for the LS of females where whole body mass was the only significant predictor (*r* = 0.53) (Fig. 3).

## Discussion

### Research aim #1

**Fig. 4:**
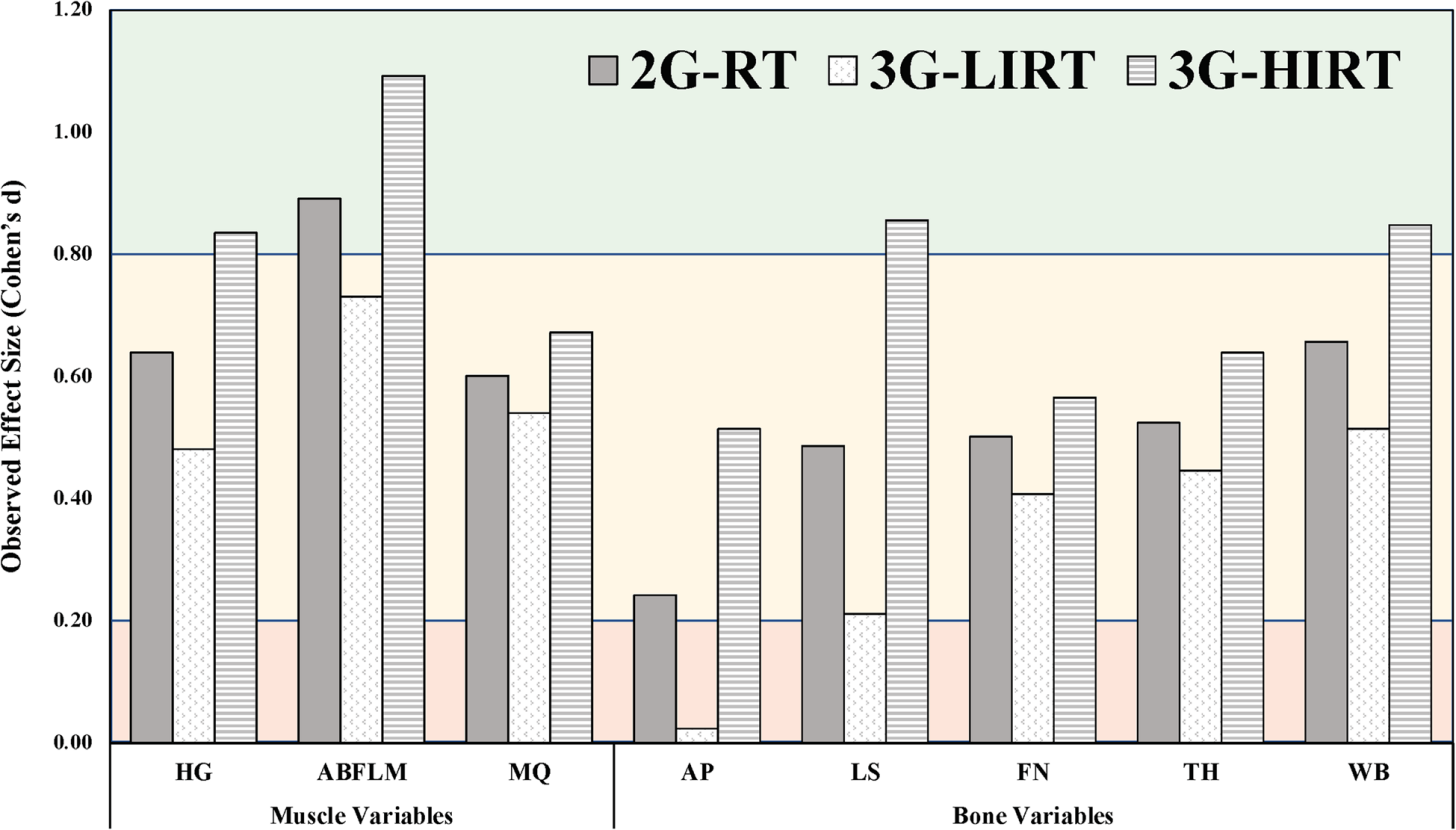
Observed effect sizes for the 2-group and 3-group methods. 2G-RT = 2 group method resistance trained compared as to NRT. 3G-LIRT = 3-group method low-intensity resistance-trained; 3G-HIRT = 3-group method high-intensity resistance-trained; HG = combined left and right maximum handgrip strength; ABFLM = combined right and left arm bone-mineral-free lean mass; MQ = muscle quality (HG/ABFLM); AP = anteroposterior bone mineral density; LS = lateral spine bone mineral density; FN = femoral neck bone mineral density; TH = total hip bone mineral density; WB = whole body bone mineral density. The background colors denote effects size: red = low, yellow = moderate, green = high

In the first research aim, we set out to describe the effect of two different classification methods of reported resistance training intensity history on variables of musculoskeletal health. These data confirm that not all self-reported resistance training is the same. The results of the 2-group and 3-group analysis reveal stark differences on the conclusions that can be made regarding the influence of one’s history with resistance training. When no consideration was given to intensity of one’s past experiences with resistance training, the data deceptively suggest that any experience with resistance training results in improved BMD. However, upon splitting the RT group into high vs. low intensity training history, improved BMD was seen for HIRT in all bone variables, with no benefits observed at any bone site for the LIRT group. A visual comparison of the 2-group and 3-group methods can be found in plotting observed effects sizes (Fig. 4). Using the 2-group method, AP BMD was not found to be significantly different between groups (*p* = 0.31), with an effect size found to be slightly above the low effect threshold of 0.24. Profoundly, when applying the 3-group method, HIRT was found to be significantly greater in AP BMD than NRT with a moderate effect size (0.52) while the LIRT was not different than NRT (*p* = 0.80) with an effect size of 0.03. Likewise, both 2- and 3-group methods reveal strength and lean mass differences between a history of resistance training and no history of resistance training. However, MQ was not found to be different in the 2-group method but was found to be significantly different in the HIRT group but not the LIRT when accounting for resistance training intensity. Differentiating between HIRT and LIRT is therefore critical in accurately characterizing the effects of resistance training on indices of muscle and bone health.

It is noteworthy that collapsing resistance training history into a single category inflates the value LIRT while masking the true benefit of HIRT. For example, in this analysis, using only a 2-group method appears to introduce false-positive (Type I) error where indices of musculoskeletal health appear to be positively related to resistance training of any intensity. After further examination, splitting the RT group into LIRT and HIRT (the 3-group method) suggests that HIRT facilitates substantial influence on musculoskeletal strength and morphology compared to NRT. Fig. 4 reveals that in a 2-group method where HIRT and LIRT are collapsed, true, large effects of HIRT conceal low (or non-existent) effects of LIRT on musculoskeletal health. Furthermore, HIRT appears to be superior to LIRT in promoting desired muscle and bone adaptation.

The major limitation with the 2-group method can be demonstrated clearly in the case of the AP BMD where lack of differentiation of resistance training intensity resulted in type II error (false negatives) and in the case of the LS, a pooled RT group produced type I error (false positives). The accurate conclusions are more likely therefore best seen in the 3-group which allows for the conclusion that resistance training is associated with meaningful osteogenesis of the spine only if it is of high-intensity. Therefore, future research designs which fail to seek differentiation of the intensity of resistance training in retrospective analyses are likely to systematically report error-prone results rife with both type I and type II error.

### Research aim #2

As per research aim #2, we set out to characterize potential differences in muscle mass, strength, MQ, and BMD in young adults due to their self-reported history of resistance training intensity. The importance of exercise and long-term resistance training supported by a diet consisting of adequate bone- and muscle- supporting nutrients such as protein, calcium, and vitamin D should be prioritized in preventing the related conditions of sarcopenia and osteoporosis [55]. This is notable in spite of the discrepant evidence suggesting self-selected resistance training intensities are lower than what is recommended for ideal musculoskeletal benefit [30]. Furthermore, the added finding that retrospectively, self-reported training status is associated with favorable musculoskeletal status is important since the holistic nature of HIRT supplants isolated target-specific interventions such as pharmacological and nutritional approaches through the added benefit of addressing multiple risk factors including strength, balance, and increased muscle mass [56, 57].

### Muscular response

The data from the 3-group method suggest that HIRT is superior or exclusively associated with beneficial musculoskeletal adaptation. For over a decade, the ACSM has advocated an evidence-based recommendation of high intensity resistance training being superior for strength development [30] with clearer evidence provided as individuals progress towards advanced states of training. The data from the present study agrees with the ACSM Position Stand as well as with more recent meta-analyses supporting HIRT for strength development across the lifespan [25, 31, 58]. Further corroboration exists with Schoenfeld et al. [25] who calculated higher effect sizes for the influence of resistance training on strength assessed as a 1RM (HIRT = 1.69; LIRT = 1.32) compared to isometric assessments (HIRT = 0.64; LIRT 0.55). The HG assessment used here, a form of isometric assessment, demonstrated HIRT and LIRT differences with observed effect size of 0.84 and 0.48 respectively as compared to the NRT group.

Conversely, hypertrophy can be seen across a spectrum of intensities. Through the adult lifespan, a spectrum of intensities will produce significant increases in muscular size in novice lifters, though moderate intensities (70–85% of RM) are recommended for experienced lifters [30, 35]. As with strength, the present study reports superior ABFLM with the HIRT group although both resistance training groups were associated with higher ABFLM that the NRT group. Potential effects of resistance training on muscle hypertrophy were not found to be intensity-specific with regards to WBFLM, a finding which is supported by a previous meta-analysis [25]. Schoenfeld et al. reported mildly higher effect size for HIRT (0.53) compared to LIRT (0.42) with regards to the ability to promote hypertrophy, while at the same time reporting insufficient evidence for concluding intensity-specific findings on whole body lean mass.

Previous research has confirmed that MQ is a dynamic phenomenon responding to acute training and recovery [59, 60], chronic training and detraining [13, 61], ageing [62–64] and disuse [65] where true differences in MQ are expected to result from neural, architectural, fiber type proportion, and contractile vs. noncontractile tissue proportion [11, 66]. With clearly elevated ABFLM in the HIRT group, quantitative changes in muscle do not fully explain the functional changes in muscle expressed via HG strength in the present study. Although higher ABFLM was seen in the LIRT group (*p* = 0.07), significant qualitative differences were only found with the HIRT. Here, MQ was found to be 5% less in the LIRT and 7.2% less in the HIRT suggesting that hypertrophy outpaces the observed increase in strength resulting in the lower MQ. An opposing finding from previous literature demonstrating increasing MQ with resistance training after 9 weeks of very high intensity (~ 87.5% of 1RM) resistance training in young and old participants [67] warrants further investigation. Ivey et al. [67] implemented a similar lean mass quantification as the present study but differed in that they assessed the quadriceps. In the present study, BFLM represents a volumetric measure of the upper extremity including both sarcomeres in series and parallel where hypertrophy of the upper arm might obscure the relationship of HG and changes in forearm cross-sectional area (CSA). With hypertrophy resulting from HIRT, volumetric increases may outpace CSA increases resulting in a systematic suppression of MQ where direct assessment of physiological CSA of the arm flexors could reveal a more accurate value of MQ. Nonetheless, future investigations are needed to explore this technique to confirm a decrease in MQ resulting from HIRT as seen in the present study.

### Skeletal response

The overall magnitude of force elicited on the skeleton has been purported to be the single most important factor in determining training-related improvements in BMD [68]. Multiple, recent meta-analyses have confirmed the benefit of resistance training as part of a mixed-training approach (along with impact training) for increasing BMD of the hip and spine in the prevention of osteoporosis [69, 70]. Likely due to a litany of factors, including inconsistencies in intensity [71], clear consensus is needed for the specific resistance training prescription for optimal gains in bone health. The preponderance of work on resistance training and bone health has been conducted in older, post-menopausal females [72]. From these works, over time, site-specificity and load-dependency effects of resistance training on BMD was introduced [33, 73] and later supported in multiple studies with young adults where higher-intensity resistance training may be more effective in eliciting changes to the spine because higher forces are necessary to promote greater gains in BMD at the FN [26]. Almstedt et al. [74] reported a 2.7–7.7% increase in BMD (depending on the skeletal site) in young males over the course of a periodized 24-week HIRT intervention with surprisingly no BMD differences found in young females. However, other research with young females using only a dynamic squat exercise with very high intensity (85–90%) has been shown effective in improving BMD at the spine (2.2%) and total hip (1%) in as little as 12 weeks [75]. Additionally, 6 months of LIRT at approximately 65–70% of 1RM, resulted in a 2–3% increase in BMD at the FN with no change in whole body or spine BMD [76]. Along with low intensity of resistance training, an alternative possible explanation of the lack of spinal response could be the utilization of a pneumatic machine-based protocol that did not axially load the vertebral column [76]. Nonetheless, the data reported by Ryan et al. [76], demonstrates that AP, LS, and FN in HIRT were all found to be significantly higher than LIRT as well as 4.5% higher in AP, 7.7% higher in LS, and 6% higher in FN compared to NRT.

It is worth mentioning that body mass is the most important predictive variable of BMD and of the components of whole body mass, BFLM is more predictive than body fat percentage, WFat, or WB mass [77]. The relevance of this relationship is key to the interpretations of the present study. Both resistance training groups displayed elevated WBFLM which in support of Ho-Pham et al. [77] is likely to be a major moderating factor in the elevated BMD of the HIRT groups.

The results of the stepwise regressions confirmed WBFLM as being linked with BMD at each bone site. The amount of explained variance seen here is supported by previous genetic studies with twins suggesting that all environmental factors (where RT would be only one) could explain 12–49% of variance in bone morphology [78, 79]. These findings collectively reinforce the understanding of connections between resistance training and musculoskeletal morphology. The degree to which HIRT a) stimulates signaling pathways of muscle and bone growth, b) promotes muscular hypertrophy which then stimulates osteogenesis, or c) some combination thereof is a question to be resolved by further prospective research. Nonetheless, this data emphasizes the importance of RT programs performed with sufficient intensity to promote muscular hypertrophy which either in turn or in conjunction aids in the development of osseous tissues.

While calcium intake could play a role in explaining the elevated BMD in the HIRT group, total vitamin D and relative protein intakes were not different across groups in the 3-group method. A significant difference was observed between HIRT and NRT where participant mean total intakes were above U.S. population means for similarly-aged males and females and were further at-or-above the recommended daily allowance (RDA) for calcium as determined by the Institute of Medicine of 1300 mg•d^− 1^ for ages 9–18 and 1000 mg•d^− 1^ for ages 19–50 [80]. Despite the differences, large variances preclude conclusions because many individual participants were on either side of the RDA threshold for calcium. Nonetheless, calcium is unlikely to explain much of the group differences in BMD because calcium intake was used as a covariate in the final analysis and the group mean intakes were near or above the RDA. All groups were similarly well below the RDA of 15 μg•d^− 1^ (600 IU) for vitamin D intake [81]. Relative protein intake was well above the RDA of 0.8 g•kg^− 1^•d^− 1^, and trended non-significantly towards higher intakes with resistance training groups, but fell below upper recommendations ranges of 1.6–1.8 g•kg^− 1^•d^− 1^ for resistance-trained individuals [82]. Because protein has been shown to be generally favorable for BMD across skeletal sites and protein values well above RDA have been suggested to be associated with favorably higher LS BMD [83], this possibly could have played a role in the results of this study.

### Limitations

By necessary design for the given first research aim, this investigation was retrospective, cross-sectional, and relied on self-reported, subjective participant response to a questionnaire. A selection bias appears as groups were assigned post hoc which prevented a random assignment and unequal group size also resulted. Therefore, the amount of objective information available regarding the training history of participants is limited. While it is plausible that HIRT explained the observed elevations in musculoskeletal mass, without prospective randomization, it is unknown to what degree participants with greater height, mass, BMI, and lean mass might have self-selected into higher intensity resistance training during adolescence.

## Conclusions

Much is known about the effects of resistance training on musculoskeletal mass and strength development from prospective, randomized, controlled trials. Primarily, this study adds to the existing literature by demonstrating the value in identifying intensity of self-reported participant history of resistance training in research investigations. This evidence shows that participant differentiation of resistance training intensity is critical in reducing potential error associated with the explainable variance of resistance training on common musculoskeletal variables. Second, this research contributes additional evidence that HIRT elicits greater musculoskeletal response than LIRT. With regards to resistance exercise training, and perhaps most importantly, high-intensity training may be imperative for stimulating increased spinal BMD in young adults prior to the attainment of PBM. Finally, in research investigations, the intensity of one’s history of resistance training must be discerned to use as a criterion in potential group assignments or as a covariate. Failure to do so may introduce systematic error due to non-homogeneity or data confounding. It is thereby recommended that any retrospective questionnaire assessing physical activity for potential baseline musculoskeletal influences must include two questions: 1) whether someone has a history of weight/resistance training and 2) whether the weight/resistance training history included high intensity training based on one-repetition maximums.

## Data Availability

Not applicable.

## Abbreviations

1RM: One-repetition maximum
ACSM: American College of Sports Medicine
AP: Anteroposterior spine
CT: Computed tomography
ABFLM: Arm bone mineral free lean mass
BFLM: Bone mineral free lean mass
BMC: Bone mineral content
BMD: Bone mineral density
DXA: Dual-energy x-ray absorptiometer
EU: European Union
FFQ: Food frequency questionnaire
FN: Femoral neck
HG: Hand grip
HIRT: High-intensity resistance training group
LIRT: Low-intensity resistance training group
LS: Lateral spine
MANCOVA: Multivariate analysis of covariance
MET: Metabolic equivalent
MQ: Muscle quality
MRI: Magnetic resonance imaging
NHANES: National Health and Nutrition Examination Survey
NRT: No history of resistance training group
NSCA: National Strength and Conditioning Association
PBM: Peak bone mass
TH: Total hip
RDA: Recommended daily allowances
RT: Resistance training
US: United States
USDA: United States Department of Agriculture
WB: Whole body
WBFLM: Whole body bone free lean mass
WFat: Whole body fat

## References

[1] Binkley N, Krueger D, Buehring B (2013). What’s in a name revisited: should osteoporosis and sarcopenia be considered components of “dysmobility syndrome?”. Osteoporos Int.

[2] Oden A, McCloskey EV, Kanis JA, Harvey NC, Johansson H (2015). Burden of high fracture probability worldwide: secular increases 2010–2040. Osteoporos Int.

[3] Cruz-Jentoft AJ, Baeyens JP, Bauer JM, Boirie Y, Cederholm T, Landi F, Martin FC, Michel J-P, Rolland Y, Schneider SM (2010). Sarcopenia: European consensus on definition and diagnosisReport of the European working group on sarcopenia in older PeopleA. J Cruz-Gentoft et al. Age Ageing.

[4] Janssen I, Shepard DS, Katzmarzyk PT, Roubenoff R (2004). The healthcare costs of sarcopenia in the United States. J Am Geriatr Soc.

[5] Kanis JA, McCloskey EV, Johansson H, Cooper C, Rizzoli R, Reginster J-Y (2013). European guidance for the diagnosis and management of osteoporosis in postmenopausal women. Osteoporos Int.

[6] Weaver C, Gordon C, Janz K, Kalkwarf H, Lappe JM, Lewis R, O’Karma M, Wallace T, Zemel B (2016). The National Osteoporosis Foundation’s position statement on peak bone mass development and lifestyle factors: a systematic review and implementation recommendations. Osteoporos Int.

[7] Frost M, Nielsen T, Brixen K, Andersen M (2015). Peak muscle mass in young men and sarcopenia in the ageing male. Osteoporos Int.

[8] Diano D, Ponti F, Guerri S, Mercatelli D, Amadori M, Gómez MPA, Battista G, Guglielmi G, Bazzocchi A (2017). Upper and lower limbs composition: a comparison between anthropometry and dual-energy X-ray absorptiometry in healthy people. Arch Osteoporos.

[9] Lexell J, Sjöström M, Nordlund AS, Taylor CC (1992). Growth and development of human muscle: a quantitative morphological study of whole vastus lateralis from childhood to adult age. Muscle Nerve.

[10] Jones EJ, Bishop PA, Woods AK, Green JM (2008). Cross-sectional area and muscular strength. Sports Med.

[11] Barbat-Artigas S, Rolland Y, Vellas B, Aubertin-Leheudre M (2013). Muscle quantity is not synonymous with muscle quality. J Am Med Dir Assoc.

[12] Reed RL, Pearlmutter L, Yochum K, Meredith KE, Mooradian AD (1991). The relationship between muscle mass and muscle strength in the elderly. J Am Geriatr Soc.

[13] Narici MV, Roi GS, Landoni L, Minetti AE, Cerretelli P (1989). Changes in force, cross-sectional area and neural activation during strength training and detraining of the human quadriceps. Eur J Appl Physiol Occup Physiol.

[14] Dodds RM, Syddall HE, Cooper R, Benzeval M, Deary IJ, Dennison EM, Der G, Gale CR, Inskip HM, Jagger C (2014). Grip strength across the life course: normative data from twelve British studies. PLoS One.

[15] Balogun JA, Akinloye AA, Adenlola SA (1991). Grip strength as a function of age, height, body weight and Quetelet index. Physiother Theory Pract.

[16] Beaudart C, Rizzoli R, Bruyère O, Reginster J-Y, Biver E (2014). Sarcopenia: burden and challenges for public health. Arch Public Health.

[17] Mankowski RT, Anton SD, Aubertin-Leheudre M (2015). The role of muscle mass, muscle quality, and body composition in risk for the metabolic syndrome and functional decline in older adults. Curr Geriatr Rep.

[18] McGregor RA, Cameron-Smith D, Poppitt SD (2014). It is not just muscle mass: a review of muscle quality, composition and metabolism during ageing as determinants of muscle function and mobility in later life. Longev Healthspan.

[19] Misic MM, Rosengren KS, Woods JA, Evans EM (2007). Muscle quality, aerobic fitness and fat mass predict lower-extremity physical function in community-dwelling older adults. Gerontology.

[20] Newman AB, Kupelian V, Visser M, Simonsick EM, Goodpaster BH, Kritchevsky SB, Tylavsky FA, Rubin SM, Harris TB (2006). Strength, but not muscle mass, is associated with mortality in the health, aging and body composition study cohort. J Gerontol A Biol Sci Med Sci.

[21] He H, Liu Y, Tian Q, Papasian C, Hu T, Deng H-W (2016). Relationship of sarcopenia and body composition with osteoporosis. Osteoporos Int.

[22] Bonjour J-P, Chevalley T, Ferrari S, Rizzoli R. Peak bone mass and its regulation. In: Pediatric Bone. 1st ed: Elsevier; 2012. p. 189–221.

[23] Faigenbaum AD, Kraemer WJ, Blimkie CJ, Jeffreys I, Micheli LJ, Nitka M, Rowland TW (2009). Youth resistance training: updated position statement paper from the national strength and conditioning association. J Strength Cond Res.

[24] Borde R, Hortobágyi T, Granacher U (2015). Dose–response relationships of resistance training in healthy old adults: a systematic review and meta-analysis. Sports Med.

[25] Schoenfeld BJ, Grgic J, Ogborn D, Krieger JW (2017). Strength and hypertrophy adaptations between low-vs. high-load resistance training: a systematic review and meta-analysis. J Strength Cond Res.

[26] Nikander R, Sievänen H, Heinonen A, Daly RM, Uusi-Rasi K, Kannus P (2010). Targeted exercise against osteoporosis: a systematic review and meta-analysis for optimising bone strength throughout life. BMC Med.

[27] Gomez-Cabello A, Ara I, González-Agüero A, Casajus J, Vicente-Rodriguez G (2012). Effects of training on bone mass in older adults. Sports Med.

[28] Steib S, Schoene D, Pfeifer K (2010). Dose-response relationship of resistance training in older adults: a meta-analysis. Med Sci Sports Exerc.

[29] Lloyd RS, Cronin JB, Faigenbaum AD, Haff GG, Howard R, Kraemer WJ, Micheli LJ, Myer GD, Oliver JL (2016). National Strength and conditioning association position statement on long-term athletic development. J Strength Cond Res.

[30] Ratamess N, Alvar B, Evetoch T, Housh T, Kibler W, Kraemer W (2009). Progression models in resistance training for healthy adults [ACSM position stand]. Med Sci Sports Exerc.

[31] Fragala MS, Cadore EL, Dorgo S, Izquierdo M, Kraemer WJ, Peterson MD, Ryan ED (2019). Resistance training for older adults: position statement from the national strength and conditioning association. J Strength Cond Res.

[32] Kohrt WM, Bloomfield SA, Little KD, Nelson ME, Yingling VR (2004). American College of Sports Medicine position stand: physical activity and bone health. Med Sci Sports Exerc.

[33] Maddalozzo GF, Snow CM (2000). High intensity resistance training: effects on bone in older men and women. Calcif Tissue Int.

[34] Gambassi BB, dos Santos MDL, FdJF A (2019). Basic guide for the application of the main variables of resistance training in elderly. Aging Clin Exp Res.

[35] Nascimento C, Ingles M, Salvador-Pascual A, Cominetti M, Gomez-Cabrera M, Viña J (2019). Sarcopenia, frailty and their prevention by exercise. Free Radic Biol Med.

[36] Hopkins WG (1991). Quantification of training in competitive sports. Sports Med.

[37] Foster JP, Carl H, Kara M, Esten PL, Brice G (2001). Differences in perceptions of training by coaches and athletes. S Afr J Sports Med.

[38] Borresen J, Lambert M (2006). Validity of self-reported training duration. Int J Sports Sci Coach.

[39] Shephard RJ (2003). Limits to the measurement of habitual physical activity by questionnaires. Br J Sports Med.

[40] LaBrie JW, Boyle S, Earle A, Almstedt HC (2018). Heavy episodic drinking is associated with poorer bone health in adolescent and young adult women. J Stud Alcohol Drugs.

[41] Pereira MA, FitzerGerald SJ, Gregg EW, Joswiak ML, Ryan WJ, Suminski RR, Utter AC, Zmuda JM (1997). A collection of physical activity questionnaires for health-related research: the aerobics center longitudinal study physical activity questionnaire. Med Sci Sports Exerc.

[42] Hartman AM, Block G, Chan W, Williams J, McAdams M, Banks WL, Robbins A (1996). Reproducibility of a self-administered diet history questionnaire administered three times over three different seasons. Nutr Cancer.

[43] Kutáč P, Bunc V, Sigmund M. Whole-body dual-energy X-ray absorptiometry demonstrates better reliability than segmental body composition analysis in college-aged students. PLoS One. 2019;14(4):e0215599. 10.1371/journal.pone.0215599 PMC6476531 31009495

[44] Guerri S, Mercatelli D, Gómez MPA, Napoli A, Battista G, Guglielmi G, Bazzocchi A (2018). Quantitative imaging techniques for the assessment of osteoporosis and sarcopenia. Quant Imaging Med Surg.

[45] Cruz-Jentoft AJ, Bahat G, Bauer J, Boirie Y, Bruyère O, Cederholm T, Cooper C, Landi F, Rolland Y, Sayer AA (2019). Writing Group for the European Working Group on sarcopenia in older people 2 (EWGSOP2), and the extended group for EWGSOP2. Sarcopenia: revised European consensus on definition and diagnosis. Age Ageing.

[46] Wind AE, Takken T, Helders PJ, Engelbert RH (2010). Is grip strength a predictor for total muscle strength in healthy children, adolescents, and young adults?. Eur J Pediatr.

[47] Innes E (1999). Handgrip strength testing: a review of the literature. Aust Occup Ther J.

[48] Gordon AM, Huxley AF, Julian FJ (1966). The variation in isometric tension with sarcomere length in vertebrate muscle fibres. J Physiol.

[49] Stevens JP (2012). Applied multivariate statistics for the social sciences: Routledge.

[50] Ghasemi A, Zahediasl S (2012). Normality tests for statistical analysis: a guide for non-statisticians. Int J Endocrinol Metab.

[51] Cauley JA, Fullman RL, Stone KL, Zmuda JM, Bauer DC, Barrett-Connor E, Ensrud K, Lau EM, Orwoll ES (2005). Factors associated with the lumbar spine and proximal femur bone mineral density in older men. Osteoporos Int.

[52] Heymsfield SB, Heo M, Thomas D, Pietrobelli A (2011). Scaling of body composition to height: relevance to height-normalized indexes. Am J Clin Nutr.

[53] Folland JP, Mc Cauley TM, Williams AG (2008). Allometric scaling of strength measurements to body size. Eur J Appl Physiol.

[54] Cohen J (1988). Statistical power analysis for the behavioral sciences.

[55] Reginster J-Y, Beaudart C, Buckinx F, Bruyère O (2016). Osteoporosis and sarcopenia: two diseases or one?. Curr Opin Clin Nutr Metab Care.

[56] Layne JE, Nelson ME (1999). The effects of progressive resistance training on bone density: a review. Med Sci Sports Exerc.

[57] Gambassi BB, Coelho-Junior HJ, Schwingel PA, Almeida FJF, Gaspar Novais TM, PdL LO, Sauaia BA, Melo CD, Uchida MC, Rodrigues B. Resistance training and stroke: a critical analysis of different training programs. Stroke Res Treat. 2017;2017:1–11. 10.1155/2017/4830265 PMC5750509 29423327

[58] Schoenfeld BJ, Wilson JM, Lowery RP, Krieger JW (2016). Muscular adaptations in low-versus high-load resistance training: a meta-analysis. Eur J Sport Sci.

[59] Nosaka K, Clarkson PM, McGuiggin ME, Byrne JM (1991). Time course of muscle adaptation after high force eccentric exercise. Eur J Appl Physiol Occup Physiol.

[60] Radaelli R, Bottaro M, Wilhelm EN, Wagner DR, Pinto RS (2012). Time course of strength and echo intensity recovery after resistance exercise in women. J Cond Strength Res.

[61] Moritani T, deVries HA (1979). Neural factors versus hypertrophy in the time course of muscle strength gain. Am J Phys Med.

[62] Delmonico MJ, Harris TB, Visser M, Park SW, Conroy MB, Velasquez-Mieyer P, Boudreau R, Manini TM, Nevitt M, Newman AB (2009). Longitudinal study of muscle strength, quality, and adipose tissue infiltration. Am J Clin Nutr.

[63] Goodpaster BH, Park SW, Harris TB, Kritchevsky SB, Nevitt M, Schwartz AV, Simonsick EM, Tylavsky FA, Visser M, Newman AB (2006). The loss of skeletal muscle strength, mass, and quality in older adults: the health, aging and body composition study. J Gerontol A Biol Sci Med Sci.

[64] Russ DW, Gregg-Cornell K, Conaway MJ, Clark BC (2012). Evolving concepts on the age-related changes in “muscle quality”. J Cachexia Sarcopeni.

[65] Berg HE, Larsson L, Tesch PA (1997). Lower limb skeletal muscle function after 6 wk of bed rest. J Appl Physiol.

[66] Akima H, Kano Y, Enomoto Y, Ishizu M, Okada M, Oishi Y, Katsuta S, Kuno S (2001). Muscle function in 164 men and women aged 20--84 yr. Med Sci Sports Exerc.

[67] Ivey F, Tracy B, Lemmer J, NessAiver M, Metter E, Fozard J, Hurley BF (2000). Effects of strength training and detraining on muscle quality: age and gender comparisons. J Gerontol A Biol Sci Med Sci.

[68] Kohrt WM, Barry DW, Schwartz RS (2009). Muscle forces or gravity: what predominates mechanical loading on bone?. Med Sci Sports Exerc.

[69] Martyn-St James M, Carroll S (2009). A meta-analysis of impact exercise on postmenopausal bone loss: the case for mixed loading exercise programmes. Br J Sports Med.

[70] Zhao R, Zhao M, Xu Z (2015). The effects of differing resistance training modes on the preservation of bone mineral density in postmenopausal women: a meta-analysis. Osteoporos Int.

[71] Cussler EC, Lohman TG, Going SB, Houtkooper LB, Metcalfe LL, Flint-Wagner HG, Harris RB, Teixeira PJ (2003). Weight lifted in strength training predicts bone change in postmenopausal women. Med Sci Sports Exerc.

[72] Westcott WL (2012). Resistance training is medicine: effects of strength training on health. Curr Sports Med Rep.

[73] Kerr D, Morton A, Dick I, Prince R (1996). Exercise effects on bone mass in postmenopausal women are site-specific and load-dependent. J Bone Miner Res.

[74] Almstedt HC, Canepa JA, Ramirez DA, Shoepe TC (2011). Changes in bone mineral density in response to 24 weeks of resistance training in college-age men and women. J Cond Strength Res.

[75] Mosti MP, Carlsen T, Aas E, Hoff J, Stunes AK, Syversen U (2014). Maximal strength training improves bone mineral density and neuromuscular performance in young adult women. J Strength Cond Res.

[76] Ryan AS, Ivey FM, Hurlbut DE, Martel GF, Lemmer JT, Sorkin JD, Metter EJ, Fleg JL, Hurley BF (2004). Regional bone mineral density after resistive training in young and older men and women. Scand J Med Sci Sports.

[77] Ho-Pham LT, Nguyen UD, Nguyen TV (2014). Association between lean mass, fat mass, and bone mineral density: a meta-analysis. J Clin Endocrinol Metab.

[78] Bjørnerem Å, Bui M, Wang X, Ghasem-Zadeh A, Hopper JL, Zebaze R, Seeman E (2015). Genetic and environmental variances of bone microarchitecture and bone remodeling markers: a twin study. J Bone Miner Res.

[79] Nguyen T, Howard G, Kelly P, Eisman JA (1998). Bone mass, lean mass, and fat mass: same genes or same environments?. Am J Epidemiol.

[80] Institute of Medicine F, and Nutrition Board (1997). Dietary reference intakes fof calcium, phosphorous, magnesium, vitamin D, and Flouride.

[81] Del Valle HB, Yaktine AL, Taylor CL, Ross AC (2011). Dietary reference intakes for calcium and vitamin D: National Academies Press.

[82] Lemon PW (2000). Beyond the zone: protein needs of active individuals. J Am Coll Nutr.

[83] Shams-White MM, Chung M, Du M, Fu Z, Insogna KL, Karlsen MC, LeBoff MS, Shapses SA, Sackey J, Wallace TC (2017). Dietary protein and bone health: a systematic review and meta-analysis from the National Osteoporosis Foundation. Am J Clin Nutr.

